# Research progress on pyroptosis regulated by mesenchymal stem cell-derived exosomes

**DOI:** 10.3389/fimmu.2025.1717465

**Published:** 2025-12-04

**Authors:** Tingyu Yang, Jiapan An, Xinqi Xu, Bin Li, Zhimin Dou

**Affiliations:** 1 The First School of Clinical Medicine, Lanzhou University, Lanzhou, China; 2 Department of Critical Care Medicine, The First Hospital of Lanzhou University, Lanzhou, China

**Keywords:** mesenchymal stem cells, exosomes, pyroptosis, pathway, gasdermin

## Abstract

Pyroptosis is a newly discovered form of inflammatory programmed cell death, which is frequently involved in the occurrence and development of various diseases. The primary mechanism underlying pyroptosis is the formation of membrane pores mediated by activated pyroptosis-related proteins. The expression levels of these pyroptosis-related proteins serve as crucial biomarkers for assessing the degree of pyroptosis. Modulating pyroptosis can alleviate tissue and organ damage in diseases and promote tissue and organ repair. Therefore, regulating pyroptosis is considered a potential therapeutic strategy. In recent years, mesenchymal stem cell-derived exosomes (MSC-Exos) have emerged as a novel therapeutic tool for pyroptosis due to their carrier properties. MSC-Exos can mitigate tissue damage in various diseases by regulating pyroptosis, thus emerging as strong candidates for disease treatment. Owing to their multifunctionality, MSC-Exos exert different effects by mediating different pathways in the treatment of various diseases. This review summarizes the mechanisms of pyroptosis and the research progress on MSC-Exos-regulated pyroptosis and outlines the existing challenges for the clinical translation of MSC-Exos. Collectively, MSC-Exos can not only precisely regulate the pyroptosis process but also provide new perspectives and approaches for future disease treatment. Therefore, MSC-Exos possess substantial potential for clinical translation.

## 1 Introduction

Pyroptosis is a form of inflammatory programmed cell death regulated by genes that can be activated in response to external stimuli and plays a significant role in the pathogenesis of various diseases (1, 2). The hallmarks of pyroptosis include pore formation in the plasma membrane, cell swelling, membrane rupture, and the release of pro-inflammatory cytokines, ultimately triggering an inflammatory response (3). Under physiological conditions, pyroptosis serves as an important defense mechanism against pathogen invasion by inhibiting intracellular pathogen replication, activating immune cells to phagocytose and kill pathogens, and releasing cellular contents as danger signals to modulate the innate immune response (4, 5). However, once pyroptosis becomes dysregulated, it can activate the inflammatory response in neighboring cells and tissues, thereby exacerbating inflammatory damage. In severe cases, this can lead to tissue damage and disease progression, adversely affecting patient prognosis (6). As a result, it is crucial to identify effective means to precisely regulate pyroptosis.

In recent years, stem cells with self-renewal and multidirectional differentiation potential have been proven to be applicable for the treatment of various diseases (7–9). Among them, mesenchymal stem cells (MSCs) have become the most commonly studied type due to their minimal ethical controversy, wide availability, and ease of acquisition (10, 11). Studies have shown that MSCs possess a variety of biological functions, including the regulation of immune responses, modulation of cell death, and promotion of tissue repair. MSCs can be used to treat a range of diseases, such as osteoarthritis, pulmonary fibrosis, bone marrow injury, myocardial injury, and knee cartilage damage (12, 13). Meanwhile, an increasing amount of evidence indicates that exosomes derived from MSCs (MSC-Exos) not only retain the therapeutic effects of the parental MSCs but also avoid the risks associated with live cell therapy (14, 15). Thus, applying MSC-Exos as a substitute for MSCs in cell-free therapy may be a focus of future research and clinical treatment.

This review aimed to elucidate the pathological mechanisms of pyroptosis, summarize the mechanisms by which MSC-Exos modulate pyroptosis and the associated signaling pathways, and review the recent research progress in this area. This effort is intended to explore the clinical application potential of MSC-Exos and provide theoretical support and practical references for future therapies targeting pyroptosis in various diseases.

## 2 Pyroptosis

Pyroptosis is a form of programmed cell death mediated by the gasdermin (GSDM) family protein. The gasdermin protein family functions as the effector molecules of pyroptosis. These proteins can be cleaved by active caspases, releasing an N-terminal domain (GSDM-NT) with pore-forming potential (16). The GSDM-NT can insert into the cell membrane and oligomerize, leading to the formation of pores with an inner diameter of approximately 15 nm on the membrane surface, thereby triggering pyroptosis (17, 18). Depending on the type of inflammatory caspase, pyroptosis can be categorized into the canonical pathway and the non-canonical pathway (19, 20).

### 2.1 Classical pyroptosis pathway

**Figure 1:**
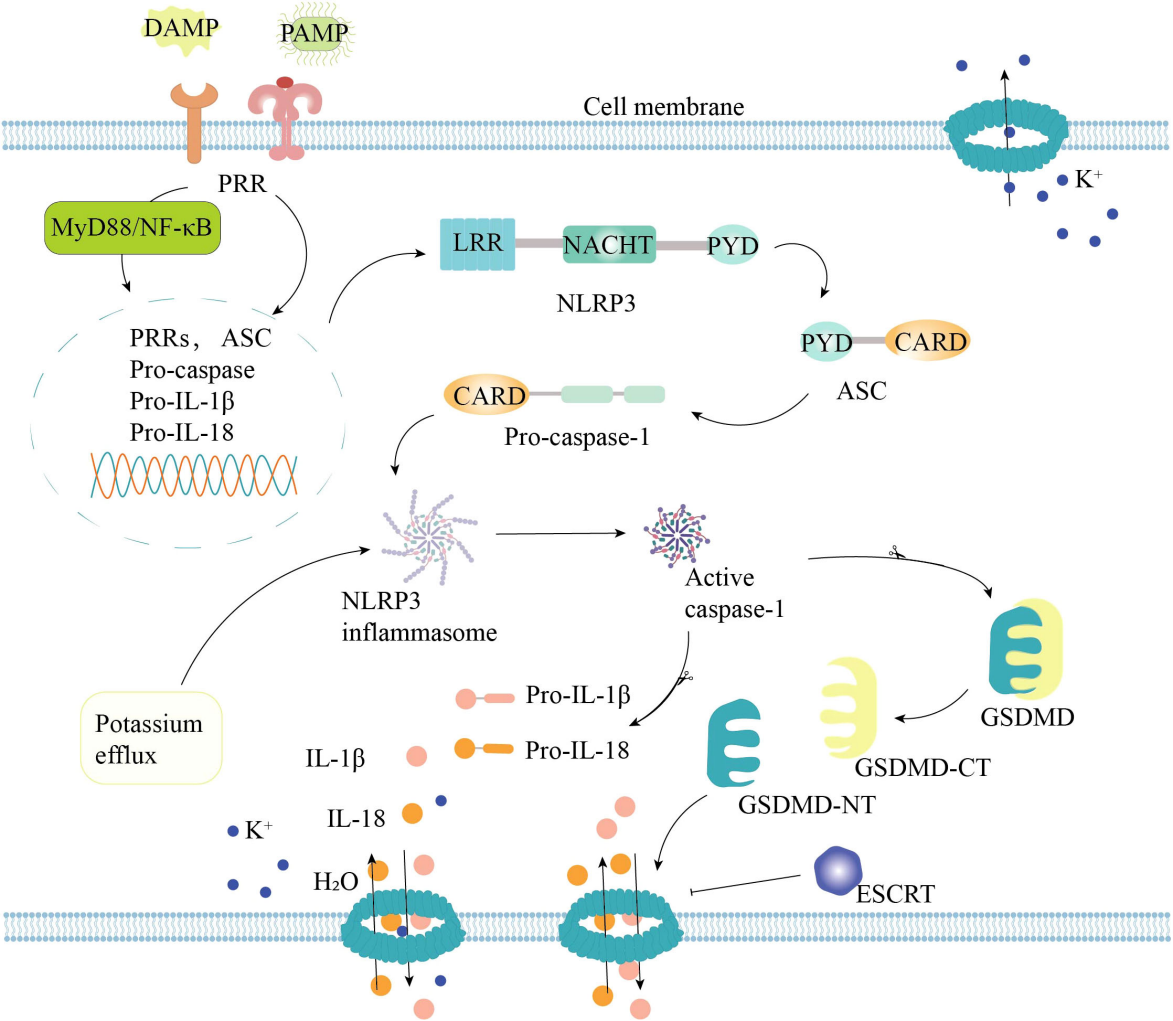
Classical pyroptosis pathway. DAMPs, pathogen-associated molecular patterns; DAMPs, damage-associated molecular patterns; PRR, pattern recognition receptors; NF-κB, nuclear factor-κB gene; ASC, apoptosis-associated speck-like protein containing CARD; PYD, PYRIN Domain; GSDM, gasdermin; ESCRT, endosomal sorting complex required for transport; IL-1β, interleukin-1β; IL-18, interleukin-18.

The classical pathway is regulated through the activation of caspase-1 by the inflammasome complex (**Figure 1**). To be specific, pattern recognition receptors (PRRs) on the cell surface can recognize a variety of pathogen-associated molecular patterns (PAMPs) and damage-associated molecular patterns (DAMPs) (21). Activated PRRs can activate the nuclear factor-κB gene (NF-κB), which in turn promotes the transcription of pyroptosis-related molecules such as nucleotide-binding oligomerization domain-like receptor 3 (NLRP3) and pro-interleukin (IL)-1β. Subsequently, apoptosis-associated speck-like protein containing a caspase recruitment domain (ASC) and pro-caspase-1 are recruited to assemble into the inflammasome (22, 23). After the formation of the inflammasome, pro-caspase-1 undergoes self-cleavage to form the active caspase-1. Active caspase-1 can cleave and remove the C-terminal domain of the GSDMD to form active GSDMD-NT, thereby triggering pyroptosis (24). Meanwhile, active caspase-1 can also cleave pro-IL-1β and pro-IL-18 to form mature IL-1β and IL-18. These inflammatory mediators can be released into the extracellular space through GSDMD pores to activate local immune cells and trigger an inflammatory storm (25, 26).

### 2.2 Non-classical pyroptosis pathway

**Figure 2:**
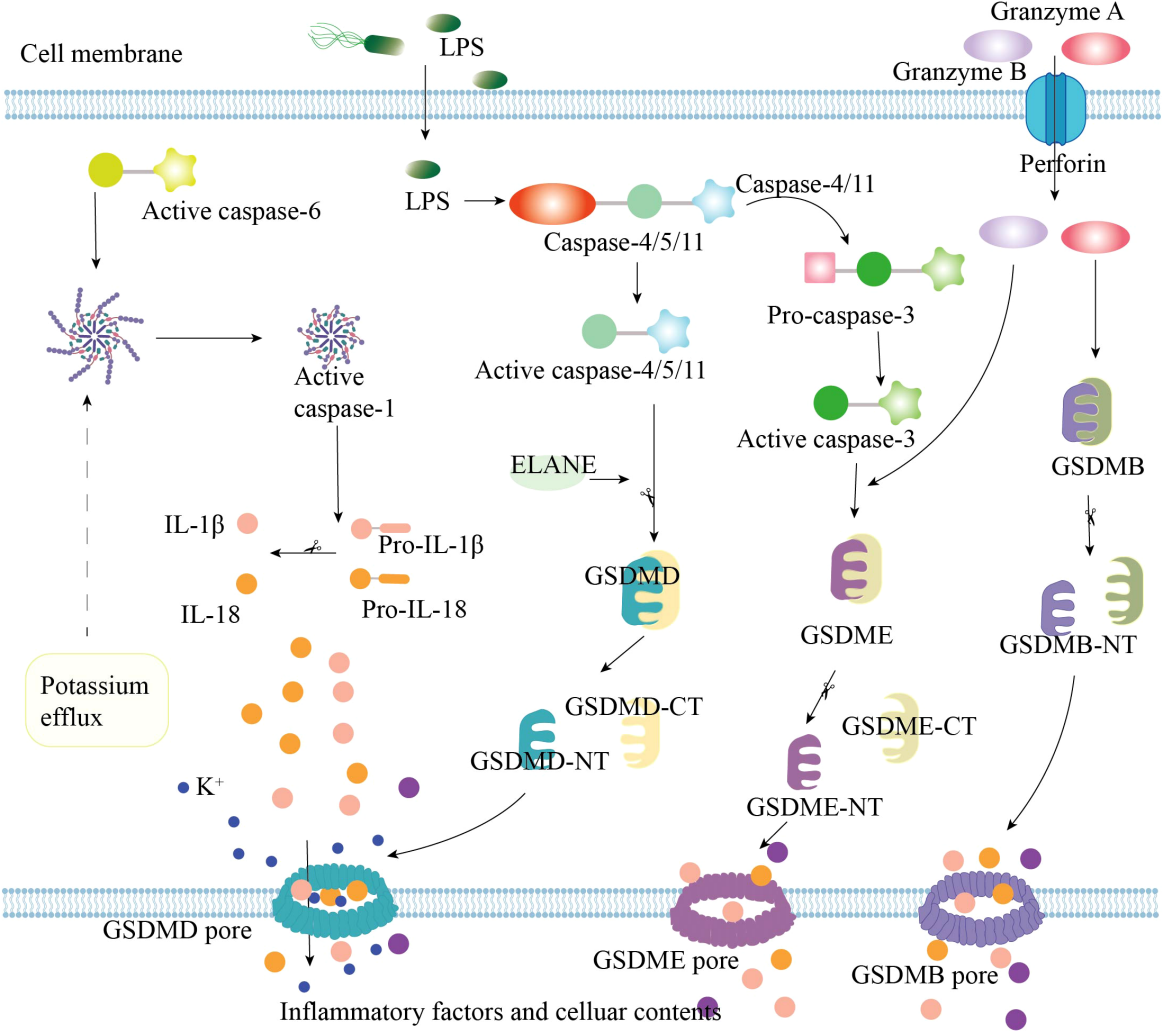
Non-classical pyroptosis pathways. LPS, lipopolysaccharide; IL-1β, interleukin-1 β; IL-18, interleukin-18; GSDM, gasdermin; ELANE, Neutrophil elastase.

The non-canonical pyroptosis pathway is directly triggered by lipopolysaccharide (LPS) and mediated by caspase-4/5/11 (**Figure 2**). Initially, LPS can enter cells through damage to the host cell membrane, bacterial outer membrane vesicles, or binding to high-mobility group box 1 (HMGB1) (27–29). Pro-caspase-4/5/11 can directly bind to intracellular LPS, inducing its own activation to produce caspase - 4/5/11 (30, 31). Active caspase-4/5/11 can cleave GSDMD into GSDMD-NT, causing pyroptosis (32–36). However, caspase-4/5/11 cannot cleave pro-IL-1β/pro-IL-18, but they can mediate the maturation and secretion of IL-1β/IL-18 by activating the NLRP3/caspase-1 pathway (37). In addition, recent studies have found that apoptotic caspases can also mediate pyroptosis (38–40). For instance, caspase-4/11 can cleave and activate pro-caspase-3, cleaving GSDME and triggering pyroptosis (41, 42). Caspase-6/8 can drive pyroptosis by modulating the activity of caspase-11 (43–46). Notably, several studies have revealed that granzyme A (GzmA) and GzmB can cleave GSDMB and GSDME, respectively, to produce active GSDMB/E-NT fragments (38, 47, 48). Neutrophil elastase is also capable of cleaving GSDMD to induce pyroptosis (39, 49).

These findings enrich our understanding of pyroptosis. It is noteworthy that recent studies have shown that the organism can remove GSDMD pores through the endosomal sorting complex required for transport (ESCRT) to inhibit pyroptosis and thus repair the plasma membrane (50). In addition, GSDMD pores can regulate the secretion of IL-1β in living cells without causing cell death (51, 52). These results suggest that pyroptosis is modulatable, providing a theoretical basis for targeting pyroptosis in disease treatment.

## 3 Overview of MSC-exos

In recent years, an increasing number of studies have demonstrated that MSCs mainly exert therapeutic effects such as anti-inflammation, immune regulation, antioxidation, and promotion of tissue injury repair through their paracrine factors, demonstrating great potential in treating various diseases (53–55). Exos, as key substances secreted by MSCs, are gradually being regarded as an alternative to stem cell therapy due to their multiple advantages, such as biocompatibility, modifiability, and non-cytotoxicity (56–59).

### 3.1 Biogenesis of exos

**Figure 3:**
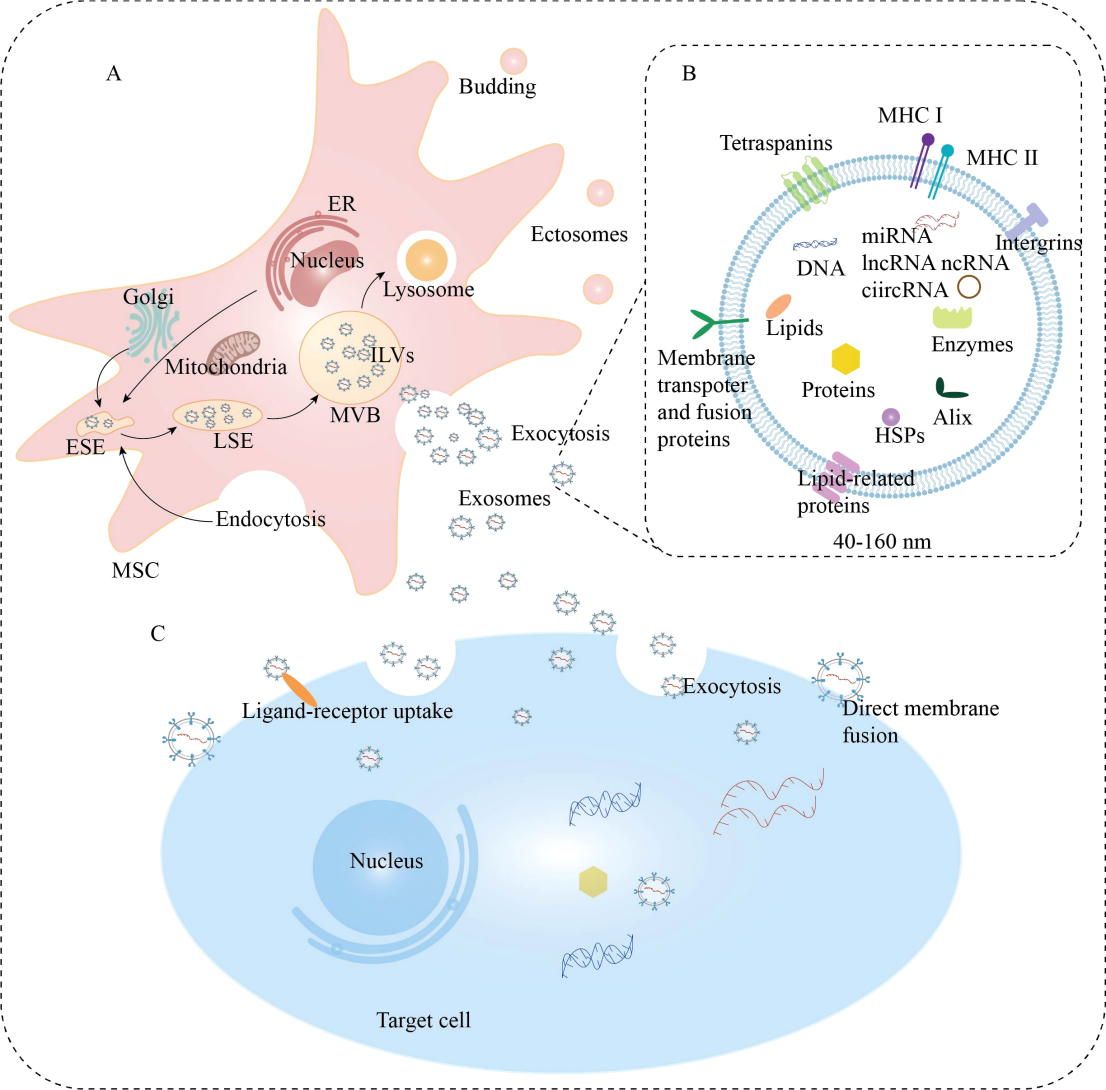
Schematic illustration of the biogenesis, compositions, and also release of the exosome. **(A)** Biogenesis of exosomes. **(B)** Compositions of exosomes. **(C)** Uptake mechanisms of exosomes by target cells. Following MVBs incorporation with the cellular membrane, the release of exosome into the extracellular space is accomplished, and finally the released molecules are conveyed to recipient cells through endocytosis, or direct membrane fusion, or receptor‐ligand interfaces. GA, Golgi apparatus; ER, endoplasmic reticulum; ESEs, early sorting endosomes; LSEs, late-stage sorting endosomes; ILVs, intraluminal vesicles; MVB, multivesicular bodies; MHC, major histocompatibility complex; HSPs, heat shock proteins.

Exosomes originate from lipid raft microdomains of the endoplasmic reticulum’s plasma membrane and are nanoscale particles with a diameter of approximately 40–160 nm (60, 61). The biogenesis of exosomes is a continuous and strictly regulated process, mainly involving two invaginations of the plasma membrane and the formation of multivesicular bodies (MVBs) (**Figure 3A**). Initially, the plasma membrane invaginates from the cell surface and the extracellular environment to encapsulate proteins and bud inward, forming early sorting endosomes (ESEs) [77, 85]. Subsequently, ESEs fuse with each other to form late sorting endosomes (LSEs). The limiting membrane of LSEs invaginates again to form MVBs [84, 86]. Ultimately, the interaction between the generated MVBs and the plasma membrane releases vesicular components, which are referred to as exosomes (62, 63). This process can be mediated by either the ESCRT pathway or non-ESCRT pathways (64–67). After secretion, exosomes facilitate intercellular communication by interacting with and fusing with recipient cells (**Figure 3C**). Additionally, various molecules, including lipids like ceramide, heat shock proteins, lactadherin, GTPases, annexins, platelet-derived growth factor receptors, and tetraspanins, play roles in the formation of exosomes (68–70).

Although we have gained some understanding of the molecules involved in the biogenesis of exosomes, it is crucial to further investigate the underlying mechanisms by which these molecules precisely regulate exosome biogenesis. This will not only enhance our ability to produce exosomes in a targeted manner but also hold significant implications for the development of cell-free therapies.

### 3.2 Isolation of exosomes

To date, there is no consensus on the “gold standard” method for exosome isolation (71). Although differential ultracentrifugation, density gradient ultracentrifugation, and tangential flow filtration have all been successfully employed to isolate exosomes, each method has its own advantages and disadvantages (72). Among them, differential high-speed ultracentrifugation is the most widely utilized and traditional method due to its simple protocol, efficiency, and high purity (73). Density gradient ultracentrifugation can isolate exosomes with higher purity than traditional ultracentrifugation by separating particles through layers of biocompatible media with different densities (74). However, none of these methods can distinguish exosomes from microvesicles and other vesicles with overlapping size ranges (75). Although capture methods based on immune affinity can differentiate various exosomes via surface markers of extracellular vesicles, their yield is too low (75, 76). In addition, in recent years, a variety of new methods have emerged, such as low-speed centrifugation based on polyethylene glycol, enrichment methods based on antibodies and filters, methods combining acoustics and microfluidics, and commercial kits (77–79). However, whether these new methods can effectively isolate exosomes has not been fully evaluated.

### 3.3 Characteristics of MSC-exos

MSC-Exos selectively package a variety of biomolecules, including proteins, mRNA, long noncoding RNA, miRNA, metabolic enzymes, and lipids (80–83) (**Figure 3B**). By facilitating the transfer of these molecules to target cells to mediate intercellular communication, exosomes exert biological effects similar to those of MSCs (84–86). Additionally, other molecules, including tetraspanins, heat shock proteins, and RNA-binding proteins, can also be packaged into exosomes, where they assist in the assembly and intracellular transport of exosomes (80, 87, 88). In addition to their inherent qualities, MSC-Exos also serve as an ideal delivery system for therapeutic compounds such as genes, drugs, enzymes, and RNA to reach specific cells (89–92). MSC-Exos have been shown to protect their cargo from degradation and promote intracellular uptake via endocytosis (93). Compared with MSCs, MSC-Exos possess a series of advantages, including low immunogenicity, high stability, preferential targeting of damaged tissues, and simple storage (94–96). Exosomes can initiate various pathophysiological responses in recipient cells, such as cell proliferation, differentiation, and development, immune regulation, homeostasis, and neurocommunication, by interacting with receptors and mediating signaling pathways (97). These advantages make them a promising cell-free alternative to existing stem cell therapies and hold great potential for disease treatment.

## 4 Effects of MSC-Exos on pyroptosis

Exosomes, as the principal mediators of the therapeutic effects of MSCs, possess inherent nanocarrier properties and contain a variety of effector molecules, including miRNAs, proteins, and long non-coding RNAs (98). A substantial body of research indicates that MSC-Exos can modulate the progression of various diseases through various mechanisms, such as influencing inflammasome activation, pyroptosis-related molecules, or the activation of specific signaling pathways, thereby exerting therapeutic effects (**Table 1**).

**Table 1:** Effects of application of mesenchymal stem cell-derived exosomes regulate pyroptosis in various disease models.

Origin	Functional molecules	Targets	Methods	Administration route/dose	Effector cells	Diseases	Effects
BMSC-exos	miR-223	ASC/caspase-1/GSDMD signalling pathway	*In vitro* and *in vivo*	IV	Tracheal epithelial cells	Asthma (104)	Improving airway remodeling and inflammation
BMSC-exos	miR-223-3p	NLRP3 mRNA	*In vitro* and *in vivo*	Local	Macrophages	RA (102)	Inhibiting inflammation and alleviating the symptoms of RA
BMSC-exos	miR-223-3p	NLRP3	*In vitro* and *in vivo*	NA	EPCs	AUI (103)	Inhibiting pyroptosis and inflammation in EPCs and promoting EPC-mediated angiogenesis
BMSC-exos	miR-223	NLRP3, caspase-1	*In vitro* and *in vivo*	IV	Hepatocytes	Autoimmune hepatitis (101)	Inhibiting the occurrence of pyroptosis and attenuating liver injury.
HucMSC-exos	miR-223-3p	NLRP3	*In vitro* and *in vivo*	IV, 200ug exosomes	Macrophages and fibroblast cells	Silicosis (105)	Inhibiting pyroptosis, decreasing inflammation, and alleviating silica-induced pulmonary fibrosis
BMSC-exos	miR-539-5p	NLRP3	*In vitro* and *in vivo*	IP, 2 × 10^10^ exosomes	Small intestinal epithelial cells	IBD (106)	Inhibiting pyroptosis and alleviating inflammatory bowel disease
ADMSC-exos	miR-22	NLRP3, caspase-1, GSDMD	*In vitro* and *in vivo*	IV, 5ug exosomes	Neurons	AD (107)	Attenuating inflammasome-related pyroptosis to improve AD
ADMSC-exos	miR-188-3p	NLRP3	*In vitro* and *in vivo*	IV 400 ug exosomes	Neurons	PD (108)	Suppressing pyroptosis of MN9D, alleviating substantia nigra damage, and ameliorating PD
ADMSC-exos	miR-26a-5p	NLRP3, caspase-1, GSDMD	*In vitro*	NA	RGCs	Diabetic retinopathy (109)	Inhibiting the pyroptosis of RGCs
HESCs-exos	miR-302c	NLRP3 inflammasome	*In vitro* and *in vivo*	Local, 0.2 ug exosomes	NPCs	IVDD (110)	Inhibiting the pyroptosis of NPCs and delaying the development of IVDD
MSC-exos	miR-410	NLRP3 mRNA	*In vitro* and *in vivo*	IV, 10 ug exosomes	NPCs	IVDD (111)	Weakening NPCs pyroptosis to alleviate IVDD
BMSC-exos	miR-182-5p	NLRP3, GSDMD	*In vitro* and *in vivo*	Local, 10 ug exosomes	Cardiomyocytes	Myocardial I/R injury (112)	Reducing inflammation and pyroptosis of cells, improving cardiac function, and decreasing the incidence of myocardial infarction
MSC-exos	miR-320b	NLRP3	*In vitro* and *in vivo*	NA	Cardiomyocytes	Myocardial I/R injury (113)	Inhibiting cardiomyocyte pyroptosis and protecting the myocardium against I/R injury
HucMSC-exos	miR-203a-3p.2	caspase-11/4	*In vitro* and *in vivo*	IV, 1000 ug exosomes	Macrophage	Colitis (114)	Relieving macrophage pyroptosis and alleviating murine colitis
HGMSC-exos	miR-128-3p, miR138-5p, and miR-221-3p	caspase-3	*In vitro*	NA	Cardiomyocytes	Myocardial I/R injury (115)	Inhibiting inflammation and cardiomyocyte pyroptosis
BMSC-exos	miR-30e-5p	caspase-1	*In vitro*	NA	human renal proximal tubular cells	DKD (116)	Inhibiting pyroptosis of human renal proximal tubular cells
BMSC-exos	miR-326	HDAC3 and STAT1/NF-κB p65 signalling pathway	*In vitro* and *in vivo*	Local, 40 ug exosomes	Chondrocytes	Osteoarthritis (120)	Inhibiting the occurrence of pyroptosis and protecting chondrocytes from osteoarthritis
BMSC-exos	miR-202-5p	CMPK2	*In vitro* and *in vivo*	IV, 100 ug exosomes	Mouse lung epithelial cells	LIRI (123)	Suppressing pyroptosis and inhibiting LIRI progression
ADMSC-exos	miR-17	TXNIP	*In vitro* and *in vivo*	IV, 400 ug exosomes	Kupffer cells	ALF (124)	Suppressing inflammasome activation to improve ALF
HGMSC-exos	miR-324-5p	NF-κB signalling pathway	*In vitro*	NA	Cardiomyocytes	Myocardial hypoxia-reperfusion injury (115)	Inhibiting inflammation and cardiomyocyte pyroptosis
HucMSC-exos	miR-100-5p	miRNA-100-5p/FOXO3/NLRP3	*In vitro*	NA	Cardiomyocytes	Myocardial I/R injury (125)	Suppressing cardiomyocytes’ pyroptosis and reducing H/R-induced injury
HucMSC-exos	miR-146a-5p	miR-146a-5/TRAF6 axis	*In vitro* and *in vivo*	Local, 5 ug exosomes	Microglia	Chronic inflammatory pain (122)	Attenuating inflammasome-related pyroptosis to improve inflammatory pain
MSC-exos	miR-140-3p	HMGB1	*In vitro* and *in vivo*	IV, 200 ug exosomes	Microglia	Sepsis-associated encephalopathy (126)	Inhibiting cellular inflammation and pyroptosis to improve cognitive impairment in mice
ADMSC-exos	miR-155-5p	TGFβR2	*In vitro* and *in vivo*	Local, 1.5×10^6^ exosomes	NPCs	IVDD (127)	Alleviating IVDD by reducing pyroptosis
BMSC-exos	miR-23b	Nrf2 signalling pathway	*In vitro* and *in vivo*	IV, 100 ug exosomes	Microglia and hippocampal neuronal cells	Cerebral haemorrhage (128)	Alleviating pyroptosis and promoting neurological recovery in rats with cerebral haemorrhage
BMSC-exos	circ-003564	Reduction of pyroptotic bodies and activation of inflammatory factors	*In vitro* and *in vivo*	IV, 200 ug exosomes	Neurons	SCI (132)	Attenuating inflammasome-related pyroptosis to improve SCI recovery
HucMSC-exos	circHIPK3	miR-421/FOXO3a signalling pathway	*In vitro* and *in vivo*	Local, 100 ug exosomes	Myoblast	Ischaemic muscle injury (133)	Inhibiting pyroptosis and inflammation in skeletal muscle cells to enhance ischemic hindlimb repair
Human MSCs-exoss	lncRNA KLF3-AS1	miR-138-5p/Sirt1 axis	*In vitro* and *in vivo*	40 ug exosomes	Cardiomyocytes	MI (134)	Attenuating pyroptosis, inflammation, and MI progression
ADMSC-exos	XIST	miR-214-3p	*In vitro* and *in vivo*	Local, 10^8^ exosomes	Cardiomyocytes	Atrial fibrillation (135)	Inhibiting inflammation and pyroptosis in cardiomyocytes

MSC, mesenchymal stem cell; ASC, apoptosis-associated speck-like protein containing a caspase recruitment domain; GSDMD, gasdermin D; NLRP3, NOD-like receptor pyrin domain containing 3; TGFβR2, transforming growth factor β receptor 2; NF-κB, nuclear factor-κB gene; HDAC3, histone deacetylase 3; STAT1, signal transducer and activator of transcription 1; CMPK2, cytidine/uridine monophosphate kinase 2; TXNIP, thioredoxin interacting protein; FOXO3, forkhead box O3; TRAF6, TNF receptor-associated factor 6; HMGB1, high mobility group box 1; Nrf2, nuclear factor erythroid 2-related factor 2; RA, Rheumatoid arthritis; HucMSCs, human umbilical cord mesenchymal stem cell; IBD, inflammatory bowel disease; AD, Alzheimer’s disease; ADMSCs, adipose‐derived mesenchymal stem cells; PD, Parkinson^’^s disease; MN9D, mouse midbrain dopaminergic neuronal cell line; RGCs, retinal ganglion cells; IVDD, intervertebral disc degeneration; HESCs, human embryonic stem cells; NPCs, nucleus pulposus cells; I/R, ischemia/reperfusion; HGMSCs, human gingival mesenchymal stem cells; DKD, diabetic kidney disease; EPCs, endothelial progenitor cells; LIRI, lung ischemia–reperfusion injury; ALF, acute liver failure; MI, Myocardial I/R injury; SCI, spinal cord injury; IV, intravenous Injection; IP, intraperitoneal; ug, microgram.

### 4.1 Pyroptosis regulation by MSC-exos-derived miRNAs

MiRNAs are a type of endogenous small non-coding RNA molecules that regulate various cellular activities, including gene expression, cell differentiation, proliferation, and apoptosis. They function by binding to complementary sequences in the 3’ untranslated region (3’ UTR) of target mRNAs, thereby promoting the translational inhibition and degradation of these mRNAs and thus regulating the expression and translation efficiency of mRNAs in target cells (99, 100). MiRNAs carried by MSC-exos can modulate pyroptosis by affecting the expression of inflammasomes and pyroptosis-related signaling pathways, thereby influencing the occurrence and development of diseases and providing new strategies and targets for the treatment of diseases.

#### 4.1.1 Regulation of inflammasome activation

MSC-Exos can deliver miRNAs that directly target inflammasome mRNAs, leading to mRNA degradation and reduced protein expression, thereby modulating gene expression and influencing the occurrence and development of diseases, providing new ideas for the treatment of pyroptosis-related diseases.

Numerous studies have shown that miR-223 in MSC-Exos can exert protective effects in various disease models by targeting and inhibiting NLRP3 (101–104). Specifically, in an ovalbumen-induced asthma rat model, MSC-Exos can deliver miR-223-3p to inhibit the formation of the NLRP3 inflammasome in tracheal epithelial cells, thereby blocking the ASC/caspase-1/GSDMD signaling pathway and reducing airway inflammation and remodeling (104). In a rheumatoid arthritis model, miR-223 in MSC-Exos can target NLRP3 mRNA in macrophages, thereby reducing the release of pro-inflammatory factors such as IL-1β, TNF-α, and IL-18 and alleviating joint damage in rats (102). In an LPS-induced acute uterine injury model, miR-223-3p from MSC-Exos can promote the degradation of NLRP3 in endothelial progenitor cells, reverse the toxic effects of LPS on endothelial progenitor cells, and improve LPS-induced acute uterine injury in mice (103). In the autoimmune hepatitis cellular model, MSC-Exos can deliver miR-223b to the target and inhibit the expression of NLRP3 and caspase-1, thereby alleviating hepatocyte damage (101). In a silicosis mouse model, miR-223-3p derived from MSC-exos can bind to circPWWP2A in macrophages, thereby targeting and inhibiting the activation of NLRP3, which leads to the alleviation of silica-induced pulmonary inflammation and fibrosis (105).

Besides, other miRNAs also possess unique regulatory characteristics. For instance, studies have shown that miR-539-5p from MSC-Exos can target NLRP3 in intestinal epithelial cells, modulating the process of pyroptosis in a mouse model of inflammatory bowel disease (IBD), thereby alleviating the progression of IBD (106). MSC-Exos carrying miR-22 can improve memory in APP/PS1 double-transgenic Alzheimer’s disease model mice by inhibiting the expression of GSDMD, NLRP3, and caspase-1 in neurons (107). MSC-Exos overexpressing miR-188-3p can inhibit autophagy and pyroptosis in Parkinson’s disease mouse and cell models by targeting CDK5 and NLRP3 (108). The miR-26a-5p derived from MSC-Exos can reduce NLRP3 inflammasome-mediated pyroptosis and alleviate high-glucose induced pyroptosis in retinal ganglion cells by suppressing the protein expression levels of NLRP3, caspase-1, and GSDMD (109). MiR-302c and miR-410 in MSC-Exos can reduce the expression of caspase-1, IL-1β, and GSDMD by inhibiting the activation of the NLRP3 inflammasome, thereby inhibiting pyroptosis of nucleus pulposus cells (NPCs) in a mouse model of intervertebral disc degeneration (110, 111). MiR-320b and miR-182-5p in MSC-Exos can negatively regulate the expression of NLRP3, reducing cell pyroptosis of cardiomyocytes caused by myocardial ischemia/reperfusion, thereby alleviating myocardial injury in animal models (112, 113).

These findings indicate that a variety of miRNAs in MSC-Exos can regulate the pathological process of cells by inhibiting inflammasome activation and blocking downstream inflammatory cascades. However, the specific mechanisms by which MSC-Exos regulate NLRP3 to inhibit pyroptosis are still not fully understood.

#### 4.1.2 Regulation of caspase family activity

The caspase family plays a crucial role as the key protease in pyroptosis execution and is responsible for cleaving the GSDM family and activating proinflammatory factors. Studies have shown that specific miRNAs can inhibit the progression of pyroptosis-related diseases by modulating the activity of caspase family proteins.

It has been found that miR-203a-3p.2 from MSC-Exos can reduce macrophage pyroptosis and thus alleviate colitis by inhibiting the activation of caspase - 11/4 in mice (114). In addition, it has been suggested that MSC-Exos can deliver miR-128-3p, miR-138-5p, and miR-221-3p to inhibit the expression of caspase-3, thereby reducing pyroptosis and inflammation in hypoxia-reperfusion myocardial cells (115). In a high-glucose-induced renal tubular epithelial cell pyroptosis model, miR-30e-5p from MSC-derived exosomes can inhibit caspase-1 activation and mediate GSDMD cleavage by targeting the RNA-binding protein embryonic lethal abnormal vision-like 1, thereby suppressing cell pyroptosis (116).

These observations demonstrate that miRNAs from MSC-Exos can also effectively inhibit cell pyroptosis by regulating the activity of the caspase family, providing new strategies and targets for the treatment of related diseases. However, the detailed mechanisms underlying miRNA regulation of caspase family activity are still not well understood.

#### 4.1.3 Regulation of pyroptosis-related signaling pathways

MSC-Exos can precisely regulate key signaling pathways such as NF-κB, PI3K/AKT, YAP/β-catenin, and STAT1 by delivering miRNAs, thereby forming a signaling network that inhibits pyroptosis (117–120).

For example, miR-326 from MSC-Exos can target HDAC3 and the STAT1/NF-κB p65 signaling pathway, inhibiting the expression of HDAC3 and NF-κB p65 while promoting the expression of STAT1, acetylated STAT1, and acetylated NF-κB p65 in chondrocytes. This process reduces the expression of pyroptosis-related proteins such as NLRP3, ASC, GSDMD, and caspase-1, thereby inhibiting chondrocyte pyroptosis and improving osteoarthritis in rats (120). Additionally, it has been found that the bioactive substances in MSC-Exos can target and regulate key upstream targets such as TRAF6, TXNIP, FOXO3, ELAVL1, and CMPK2 (116, 121–123). For example, both *in vitro* and *in vivo* studies have revealed that miR-202-5p from MSC-Exos can target and inhibit CMPK2 in alveolar epithelial cells, thereby suppressing the expression of NLRP3 and inhibiting the progression of lung ischemia-reperfusion injury (123). MiR-17 in MSC-Exos can inhibit the activation of the inflammasome in hepatic macrophages by targeting TXNIP, thereby reducing liver damage in a mouse model of acute liver failure (124). MiR-324-5p in MSC-Exos can inhibit the expression of pyroptosis-related proteins by suppressing the NF-κB signaling pathway, alleviating hypoxia-reperfusion injury to cardiomyocytes (115). MiR-100-5p in MSC-Exos can inhibit pyroptosis-related proteins by targeting the miR-100-5p/FOXO3/NLRP3 pathway, thereby reducing pyroptosis in hypoxia-reperfusion cardiomyocytes (125). MiR-146a-5p derived from MSC-Exos targets and inhibits TRAF6, thereby reducing pyroptosis of microglia in mouse models of chronic inflammatory pain. This intervention effectively diminishes neuroinflammation and alleviates inflammatory pain (122). MiR-140-3p delivered by MSC-Exos targets HMGB1 to modulate S-lactoylglutathione metabolism, thereby inhibiting pyroptosis and inflammatory responses in microglia induced by LPS. This mechanism alleviates sepsis-associated brain injury in mice (126). MiR-155-5p from MSC-Exos can promote autophagy and inhibit pyroptosis by targeting TGFβR2 in NPCs, thereby alleviating intervertebral disc degeneration in mice (127). MSC-Exos can modulate the nuclear factor erythroid 2-related factor 2 signaling pathway by delivering miR-23 b and targeting PTEN in microglia to inhibit the activation of NLRP3, ultimately alleviating pyroptosis and promoting neurological recovery in rats with cerebral hemorrhage (128). Exosomes carrying miR-367-3p derived from MSCs can inhibit muscle pyroptosis in a mouse model of hindlimb ischemia-reperfusion (I/R) injury by targeting the enhancer of zeste homolog 2 (129).

The above studies have elaborated in detail the specific pathways by which MSC-Exos regulate pyroptosis by delivering internal miRNAs, not only providing new ideas for the treatment of related diseases but also offering directions for subsequent therapeutic research.

### 4.2 Other molecular components in MSC-exos regulate pyroptosis

Apart from the extensively studied miRNAs, other types of RNAs present in MSC-Exos also have the ability to inhibit pyroptosis.

CircRNA, a newly discovered non-coding RNA, forms a circular structure through backsplicing and has remained conserved during evolution. Current research has linked it to various disease processes (130, 131). For example, a recent study reported that circ-003564 from MSC-Exos can alleviate pyroptosis in spinal cord neurons and mitigate spinal cord injury in rats by suppressing the activation of the pyroptosome and inflammatory factors (132). CircHIPK3 from MSC-Exos targeted the miR-421/FOXO3a pathway in myoblasts. By downregulating miR-421 and increasing the expression of FOXO3a, it reduces pyroptosis and repairs ischemic muscle injury in mice (133).

Moreover, lncRNAs, a non-coding RNA molecule longer than 200 nucleotides, can regulate gene expression by interacting with miRNA, mRNA, or proteins. Research indicates that lncRNA KLF3-AS1 derived from MSC-Exos can modulate the miR-138-5p/Sirt1 axis, inhibiting pyroptosis in cardiomyocytes, thereby slowing the progression of myocardial infarction in rats (134). Derived from MSC-Exos, the lncRNA XIST is capable of interacting with miR-214-3p to relieve the inhibitory effect of miR-214-3p on Arl2, thereby reducing pyroptosis of cardiomyocytes induced by atrial fibrillation and alleviating cardiac injury in a mouse model (135).

Although research on the regulation of pyroptosis by MSC-Exos is limited, existing studies have sufficiently demonstrated that MSC-exos can alleviate diseases by modulating pyroptosis-related pathways. A deeper investigation into these regulatory mechanisms will enhance our understanding of the roles of MSC-Exos and the mechanisms of diseases, while providing a theoretical basis and practical references for targeting pyroptosis in disease treatment.

## 5 Critical gaps and challenges

Despite the promising therapeutic potential of MSC-Exos in modulating pyroptosis, several critical gaps and challenges must be addressed before their clinical application.

### 5.1 Identification of pyroptosis

Although pyroptosis can be distinguished from other modes of programmed cell death, such as apoptosis and necrosis, based on its unique morphological characteristics, key molecular events, and associated inflammatory responses, the complex interplay among programmed cell death pathways means that no single identification method is absolutely specific. For instance, studies have revealed that caspase-1/8 exhibits functional pleiotropy, capable of inducing not only pyroptosis but also apoptosis. Moreover, the morphological similarities at the terminal stages of different death modalities further complicate the identification process (136). Therefore, it is currently not feasible to rely solely on any single indicator to confirm pyroptosis.

In basic research, a multifaceted approach is typically employed to assess the occurrence of pyroptosis. This includes microscopic observation of cellular morphological changes, molecular biological detection of caspase-1 activation, and the activation of the pyroptosis execution protein GSDMD, as well as the detection of mature IL-1β and IL-18 release in the cell supernatant (136). Only through this multi-angular, corroborative method can a rigorous and reliable identification of pyroptosis be achieved.

However, this multi-step, multi-technological identification method is not only technically complex but also costly. Additionally, although the cleavage of GSDMD, the activation of the NLRP3 inflammasome, and the release of IL-1β/IL-18 are important markers of pyroptosis, the specificity and sensitivity of these markers still pose certain challenges. The heterogeneity of diseases further increases the complexity of marker identification. Thus, the precise and efficient identification of pyroptosis remains an urgent challenge that needs to be addressed.

### 5.2 MSC-exos heterogeneity

The heterogeneity of MSCs-Exos may be conceptualized based on their size, content, and particularly cellular origin (80). Based on the refined classification of extracellular vesicles, exosomes contain subpopulations defined by a distinct size range (137). Size heterogeneity could be due to uneven invagination of the limiting membrane of MVBs, leading to different total amounts of material within the vesicles, or because the isolation process includes other types of vesicles (138–140). The microenvironment of cells and their inherent biological properties may also influence the types and amounts of exosome contents and potentially affect the surface biological markers of exosomes. Therefore, as observed in the analysis of miRNA content within exosomes, not all exosomes contain a similar abundance of a given molecule (141).

Moreover, the effects of exosomes on target cells can vary due to the different expression of cell surface receptors. In different target cell types, this functional heterogeneity can result in the coexistence of exosome-induced cell survival, apoptosis, and immunomodulation. Heterogeneity can also be based on the organ and tissue of origin of the exosomes. Exosomes secreted by MSCs from different tissues carry distinct biological components, endowing exosomes with unique biological functions (142). For example, exosomes secreted by MSCs derived from adipose tissue have weaker immunomodulatory properties (143), while exosomes from MSCs derived from umbilical cord tissue have stronger renewal capabilities and more effective gene transfection (144). The combination of all these features has the potential to increase the complexity of exosome-based therapies.

### 5.3 Insufficient *in vivo* validation

Over the past few years, the therapeutic potential of MSC-Exos has been validated through animal experiments in the majority of studies (**Table 1**), and their safety and potential efficacy have been demonstrated in a few reported clinical studies (**Table 2**) (145). However, given the complexity of human disease mechanisms, which may influence the activity and function of exosomes, the safety and efficacy of exosomes in disease treatment still require further *in vivo* experiments, particularly in large animal models and clinical studies, to confirm the application effects of MSC-Exos in various diseases.

**Table 2:** Ongoing clinical studies of mesenchymal stem cell-derived exosomes.

Registration ID	Title	Phase	Number of patients	Study type	Origin	Source	Route of administration	Dose
NCT07146087	Allogeneic Mesenchymal Stem Cell-Derived Exosome Therapy for Progressive Multiple Sclerosis (153)	1	20	Interventional	N/A	Allogeneic	IV	N/A
NCT02138331	Effect of Microvesicles and Exosomes Therapy on β-cell Mass in Type I Diabetes Mellitus (T1D) (154)	2/3	N/A	Interventional open-label	UC	Allogeneic	IV	N/A
NCT03437759	MSC-Exos Promote Healing of MHs (155)	1	N/A	Randomized	UC	Allogeneic	Local	50 ug/20 ug
NCT03384433	Allogeneic Mesenchymal Stem Cell-Derived Exosome in Patients with Acute Ischemic Stroke (156)	1/2	5	Randomized, single-blinded, placebo-controlled	BM	Allogeneic	Stereotaxis/intraparenchymal	200 ug
NCT04276987	A Pilot Clinical Study on Inhalation of Mesenchymal Stem Cells Exosomes Treating Severe Novel Coronavirus Pneumonia (157)	1	24	Pilot/open-label	AD	Allogeneic	Aerosol inhalation	2 × 10^8^ particles, 5 times
NCT04491240	Evaluation of Safety and Efficiency of Method of Exosome Inhalation in SARS-CoV-2 Associated Pneumonia. (COVID-19EXO) (158)	1/2	30	Interventional	N/A	Allogeneic	Inhalation	(0.5-2) × 10^10^ particles, twice a day for 10 days,
NCT04356300	Multiple Organ Dysfunction Syndrome After Surgical Repair of Acute Type A Aortic Dissection (159)	N/A	60	Interventional	N/A	Autologous	IV	150 mg once a day for 14 times
NCT04213248	Effect of UC-MSC-Exos on Dry Eye in Patients With cGVHD (160)	1/2	27	Single group assignment	UC	Allogeneic	Artificial tears	10 ug, four times a day for 14 days
NCT04388982	The Safety and the Efficacy Evaluation of Allogeneic Adipose MSC-Exos in Patients with Alzheimer’s Disease (161)	1/2	9	Non-randomized	AD	Allogeneic	Nasal drip	5/10/20 ug twice a week for 12 weeks
NCT04173650	MSC-EVs in Dystrophic Epidermolysis Bullosa (162)	N/A	10	Single group assignment	BM	Allogeneic	Local	N/A
NCT04602104	A Clinical Study of Mesenchymal Stem Cell Exosomes Nebulizer for the Treatment of ARDS (163)	1/2	169	Randomized, double-blinded, controlled	N/A	Allogeneic	Inhalation	2/8/16× 10^8^ particles, once daily for a week
NCT04544215	A Clinical Study of Mesenchymal Progenitor Cell Exosomes Nebulizer for the Treatment of Pulmonary Infection (164)	1/2	60	Randomized, double-blinded, controlled	AD	Allogeneic	Inhalation	8/16× 10^8^ particles, once daily for a week
NCT04270006	Evaluation of Adipose Derived Stem Cells Exo.in Treatment of Periodontitis (exosomes) (165)	1	10	Open label	AD	Autologous	Local	N/A
NCT03608631	iExosomes in Treating Participants with Metastatic Pancreas Cancer with KrasG12D Mutation (166)	1	28	Open label	N/A	Allogeneic	IV	N/A
NCT05060107	Intra-articular Injection of MSC-derived Exosomes in Knee Osteoarthritis (167)	1	10	Open label	N/A	Allogeneic	IA	(3-5) × 10e11 particles
NCT04657458	Expanded Access for Use of bmMSC-Derived Extracellular Vesicles in Patients With COVID-19 Associated ARDSs (168)	N/A	200	Prospective non-randomized open-label cohort study	BM	Allogeneic	IV	N/A

MSC, mesenchymal stem cell; EVs, extracellular Vesicles; GvHD: Graft-versus-host disease; UC, umbilical cord tissue; AD, adipose tissue; BM, bone marrow; IV, intravenous Injection; IA, intra-arterial; ARDS, acute respiratory distress syndrome; ug, microgram.

Data were sourced from the clinical trials registry at https://clinicaltrials.gov/.

### 5.4 Standardization of therapeutic applications

Although MSCs can be isolated from various tissues, in current basic experiments and registered clinical trials, MSCs derived from adipose tissue, bone marrow, or umbilical cord are commonly used as sources of exosomes. Among these, bone marrow is the most frequently used source of MSC-Exos in basic research (62), while adipose tissue is the most commonly used source in clinical research (**Tables 2**, **3**). Given the non-immunogenic nature of MSC-Exos, allogeneic sources have been used in all but two studies. In addition, the routes of administration for MSC-Exos in existing studies encompass intravenous infusion, inhalation, or local delivery (**Tables 1**–**3**). The majority of these studies employ intravenous infusion or inhalation as the administration routes. Consistent with the basic research (**Table 1**), the dosing regimens in these clinical studies vary according to the route of administration and the specific disease being treated. Moreover, the units used to quantify MSC-Exos also differ: some studies measure the quantity of MSC-Exos by weight (in micrograms), others by particle number, while some merely specify the number of MSCs used to generate the MSC-Exos. These inconsistencies highlight the lack of consensus on the application of MSC-Exos, rendering it impossible to conduct a robust assessment of their therapeutic efficacy. Therefore, standardizing the application of exosomes across different diseases remains a significant challenge at present.

**Table 3:** Published clinical studies of mesenchymal stem cell-derived exosomes.

Registration ID	Title	Phase	Number of patients	Study type	Origin	Source	Route of administration	Dose	Effects
N/A	Umbilical cord mesenchymal stem cells derived extracellular vesicles can safely ameliorate the progression of chronic kidney diseases (169)	2/3	40	Single-center, randomized, placebo-controlled, phase II/III clinical pilot study	UC	Allogeneic	IV/IA	100 ug/kg	Significant improvement in eGFR, serum creatinine level, blood urea, and UACR
N/A	Skin Brightening Efficacy of Exosomes Derived from Human Adipose Tissue-Derived Stem/Stromal Cells: A Prospective, Split-Face, Randomized Placebo-Controlled Study (170)	N/A	21	A prospective, split-face, randomized, placebo-controlled study	AD	Allogeneic	Local	0.2 g, twice a day for 8 weeks	Significantly reduced the amount of melanin for 2 months
N/A	Combination Treatment with Human Adipose Tissue Stem Cell-derived Exosomes and Fractional CO2 Laser for Acne Scars: A 12-week Prospective, Double-blind, Randomized, Split-face Study (171)	N/A	25	N/A	AD	Allogeneic	Local	9.78× 10^10^ particles	Reduced the size of skin pores and skin surface scabrously from baseline on the treated side
N/A	IRB Approved Pilot Safety Study of an Extracellular Vesicle Isolate Product Evaluating the Treatment of Osteoarthritis in Combat-Related Injuries (172)	N/A	33	Pilot study	BM	Allogeneic	Local	Exosomes produced from 1–10 × 10^6^ MSCs/kg	After a six-month follow-up, the average patient improved
NCT04313647	A Tolerance Clinical Study on Aerosol Inhalation of Mesenchymal Stem Cells Exosomes in Healthy Volunteers (173)	1/2	24	Non-randomized/open-label	AD	Allogeneic	Inhalation	2 × 10^8^ to 16 × 10^8^ particles	MSC-Exos were safe; the volunteers tolerated the infusion well and did not show adverse reactions within the week after nebulization.

GvHD, Graft-versus-host disease; UC, umbilical cord tissue; AD, adipose tissue; BM, bone marrow; IV, intravenous Injection; IA, intra-arterial; ug, microgram; kg, kilogram; g, gram.

Data were sourced from the clinical trials registry at https://clinicaltrials.gov/and https://www.google.com/.

## 6 Conclusions and prospects

Pyroptosis is a form of programmed cell death whose mechanism involves the activation of caspases and the GSDM family, as well as the release of inflammatory factors. These key steps will trigger cell death and severe inflammatory responses, ultimately leading to the progression of various diseases (146). Therefore, it is essential to identify key biomarkers for pyroptosis and to seek therapeutic strategies to modulate this process (147).

**Figure 4:**
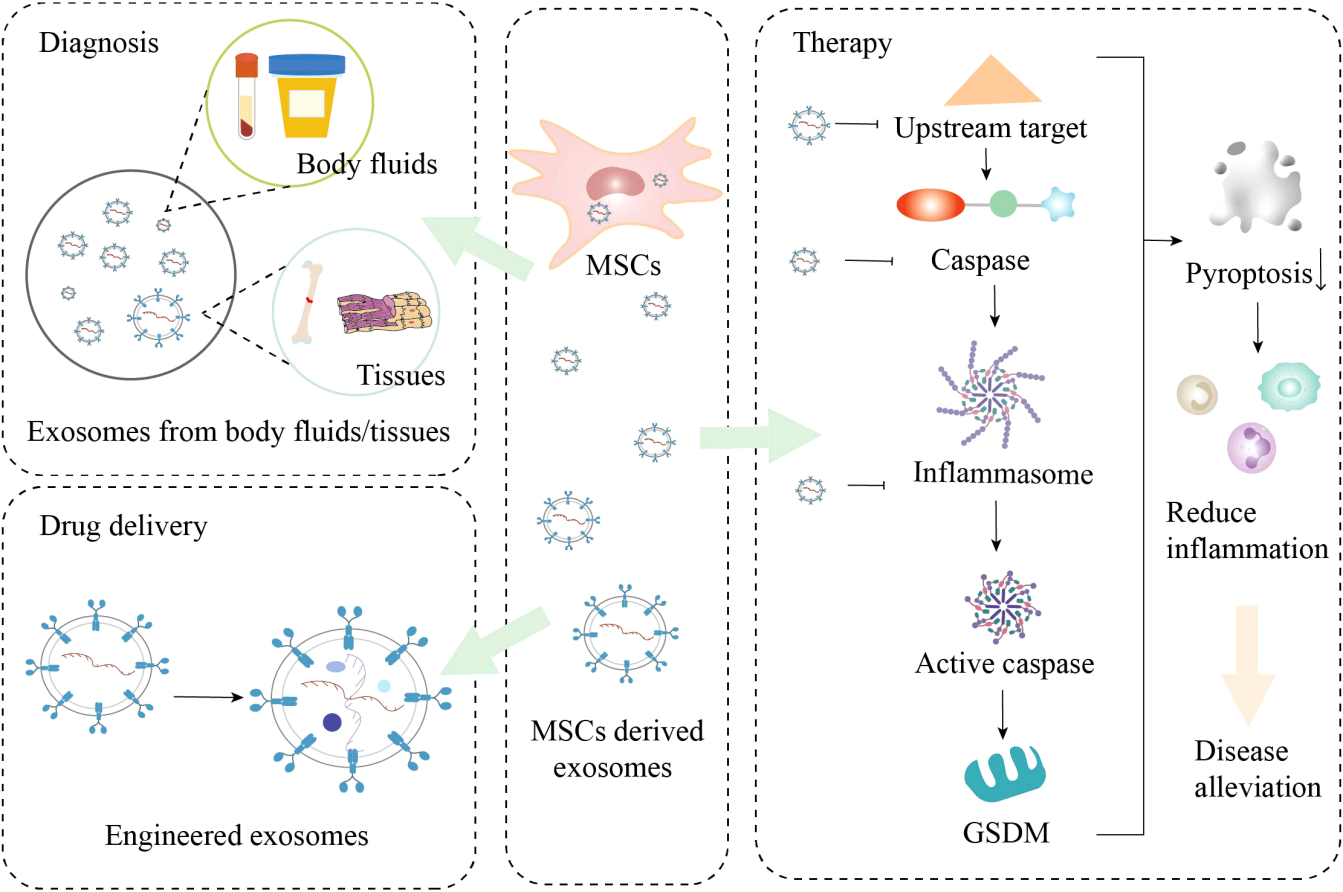
Applications of mesenchymal stem cells (MSCs) derived exosomes. GSDM, gasdermin.

MSCs have shown great potential in the treatment of various diseases due to their multidifferentiation, self-renewal, and immunomodulatory properties. Exosomes derived from MSCs not only retain the therapeutic functions of the parental cells but also possess characteristics such as non-proliferative capacity, low immunogenicity, and the capacity for cargo loading and targeted delivery (148). These characteristics make exosomes a strong candidate for replacing MSCs in disease diagnosis and treatment (**Figure 4**).

In terms of diagnostic potential, MSC-Exos may contain differentially expressed miRNAs, ncRNAs, and proteins under various disease conditions. By extracting exosomes from diseased individuals and identifying the differential expression of specific substances within them, a highly sensitive method for the identification and monitoring of pyroptosis in diseases may be provided. These substances may help accurately reflect the state and extent of pyroptosis in diseases, thereby improving the precision of pyroptosis identification. This will render exosomes a promising candidate for early pyroptosis detection, monitoring, and the development of personalized therapeutic strategies.

From a therapeutic perspective, MSC-Exos hold distinct advantages over traditional drugs. Traditional drugs exert broad-spectrum effects on the entire body, whereas MSC-Exos can selectively target specific cells and tissues, offering more precise and manageable therapeutic options. Moreover, exosomes, characterized by their low toxicity, weak immunogenicity, and high permeability, can serve as efficient delivery vehicles for substances such as miRNAs, enzymes, and proteins. The lipid bilayer membrane of exosomes protects the encapsulated substances, ensuring their stability and reducing their susceptibility to enzymatic degradation. These attributes render MSC-Exos a potentially more practical therapeutic strategy. Emerging evidence indicates that MSC-Exos can target and modulate key nodes in the pyroptosis pathways to inhibit pyroptosis, thus becoming a research focus in regenerative medicine.

Over the past few years, with the progress of genetic engineering and nanotechnology, MSC-Exos have gradually emerged as an alternative strategy to stem cell-based regenerative therapies. Exosomes can be genetically engineered to deliver various therapeutic components to the desired targets (149). Through genetic engineering and chemical modification, the targeting ability of exosomes can be enhanced, and their circulation half-life in the body can be extended, thereby improving their therapeutic efficacy (150). Compared with traditional exogenous nanocarriers, engineered exosomes possess high bioavailability, low toxicity, drug protection, and precise targeting capabilities, making them one of the most promising drug delivery vehicles currently available. Moreover, the adoption of combination therapy strategies may also aid in disease treatment. Studies have shown that the therapeutic efficacy of single MSC-Exos can be enhanced through co-culture, combined drugs, combined physical factors, and genetic modification (151, 152).

In summary, MSC-Exos have the potential to alleviate disease progression by modulating pyroptosis, thus offering a promising therapeutic avenue. However, the clinical translation of MSC-exos therapy faces several challenges. Nevertheless, with the advancement of research, the clinical potential of MSC-exos is expected to be enhanced through several approaches: elucidating the specific biomarkers, key targets, and signaling pathways of pyroptosis in various diseases; optimizing the tissue sources, production techniques, and administration strategies of MSC-exos; and developing engineered exosomes with specificity and combination therapies. In the future, MSC-exos are anticipated to serve as a tool for early detection, precise monitoring, and targeted modulation of pyroptosis, thereby treating diseases and preventing disease progression, offering new therapeutic options for patients.

## Glossary

MSCs: mesenchymal stem cells
MSC-Exos: exosomes derived from MSCs
GSDM: gasdermin
GSDM-NT: gasdermin N-terminal fragment
PRRs: pattern recognition receptors
PAMPs: pathogen-associated molecular patterns
DAMPs: damage-associated molecular patterns
NLRP3: NOD-like receptor pyrin domain containing 3
IL-1β: interleukin-1β
NF-κB: nuclear factor-κB gene
ASC: apoptosis-associated speck-like protein containing a caspase recruitment domain
LPS: lipopolysaccharide
HMGB1: high-mobility group box 1
ZBP1: Z-DNA Binding Protein 1
TNF: tumor necrosis factor
Gzm: granzyme
ESCRT: endosomal sorting complex required for transport
ESEs: early sorting endosomes
LSEs: late sorting endosomes
MVBs: multivesicular bodies
NPCs: nucleus pulposus cells.
